# Spatiotemporal bayesian modelling of scorpionism and its risk factors in the state of São Paulo, Brazil

**DOI:** 10.1371/journal.pntd.0011435

**Published:** 2023-06-20

**Authors:** Francisco Chiaravalloti-Neto, Camila Lorenz, Alec Brian Lacerda, Thiago Salomão de Azevedo, Denise Maria Cândido, Luciano José Eloy, Fan Hui Wen, Marta Blangiardo, Monica Pirani

**Affiliations:** 1 School of Public Health, University of São Paulo, São Paulo, Brazil; 2 Health Department of the Municipality of Santa Bárbara d’Oeste, São Paulo, Brazil; 3 Instituto Butantan, São Paulo, Brazil; 4 Epidemiological Surveillance Center “Prof. Alexandre Vranjac”, São Paulo, Brazil; 5 MRC Centre for Environment & Health, Department of Epidemiology and Biostatistics, School of Public Health, Imperial College London, London, United Kingdom; Instituto Butantan, BRAZIL

## Abstract

**Background:**

Scorpion stings in Brazil represent a major public health problem due to their incidence and their potential ability to lead to severe and often fatal clinical outcomes. A better understanding of scorpionism determinants is essential for a precise comprehension of accident dynamics and to guide public policy. Our study is the first to model the spatio-temporal variability of scorpionism across municipalities in São Paulo (SP) and to investigate its relationship with demographic, socioeconomic, environmental, and climatic variables.

**Methodology:**

This ecological study analyzed secondary data on scorpion envenomation in SP from 2008 to 2021, using the Integrated Nested Laplace Approximation (INLA) to perform Bayesian inference for detection of areas and periods with the most suitable conditions for scorpionism.

**Principal findings:**

From the spring of 2008 to 2021, the relative risk (RR) increased eight times in SP, from 0.47 (95%CI 0.43–0.51) to 3.57 (95%CI 3.36–3.78), although there has been an apparent stabilization since 2019. The western, northern, and northwestern parts of SP showed higher risks; overall, there was a 13% decrease in scorpionism during winters. Among the covariates considered, an increase of one standard deviation in the Gini index, which captures income inequality, was associated with a 11% increase in scorpion envenomation. Maximum temperatures were also associated with scorpionism, with risks doubling for temperatures above 36°C. Relative humidity displayed a nonlinear association, with a 50% increase in risk for 30–32% humidity and reached a minimum of 0.63 RR for 75–76% humidity.

**Conclusions:**

Higher temperatures, lower humidity, and social inequalities were associated with a higher risk of scorpionism in SP municipalities. By capturing local and temporal relationships across space and time, authorities can design more effective strategies that adhere to local and temporal considerations.

## Author summary

Scorpions cause approximately 3,000 deaths every year worldwide, second only to snakebites in terms of venomous animal-related human accidents due to the potency of their toxins. Natural habitat extinction combined with accelerated urban expansion have promoted greater contact between humans and many species of wild animals, and consequently increased the number of accidents and fatalities caused by these animals. Recognizing which environmental conditions are most associated with accident hotspots can help design better strategies for the control and distribution of anti-scorpion serum, for example. Here we show that scorpionism occurred more frequently in spring, and it decreased in winter. The areas with higher temperatures, lower humidity, and social inequalities were also associated with a higher risk of scorpion envenomation.

## Introduction

Accidents caused by venomous animals pose a threat to public health. Among these accidents, those provoked by scorpions are becoming more frequent in many parts of the world. Owing to their high incidence and lethality, these fatalities are currently considered a global medical-sanitary threat [1]. Approximately two billion people live in regions at risk for scorpion stings worldwide, and each year an estimated 1.2 million people are victims of scorpion stings. Due to the potency of their toxins, scorpions cause approximately 3,000 deaths every year, second only to snakebites in terms of venomous animal-related human accidents [2]. Clinical outcomes caused by scorpion toxins are known as scorpionism or scorpion envenomation [1,3].

Over the last decade, there has been a wide increase in scorpionism reports, particularly in Brazil, representing a leading cause of fatalities caused by venomous animals in the country [3]. The main genus of scorpions of medical importance found in Brazil is *Tityus*, and *Tityus serrulatus* (yellow scorpion) is the most dangerous species, causing many fatalities [4]. Due to its high and rapid proliferation–and good adaptation to urban environments–it is usually found in all Brazilian regions [4]. *Tityus serrulatus* are typically nocturnal creatures and can be located in a variety of habitats, including rock crevices, tree bark, decaying tree trunks, beneath rocks and leaves, and within burrows and caves. The presence and abundance of these scorpions in a given area is largely dependent on factors such as temperature, humidity, and the availability of prey [4].

Natural habitat extinction combined with accelerated urban expansion have promoted greater contact between humans and wild animals of many species, and consequently increased the number of accidents and fatalities caused by scorpions [1,5,6]. Recognition of priority areas for public health actions is crucial for effective decision-making in conditions that require allocation of limited resources (e.g., anti-scorpion serum and patient healthcare). Spatial analysis is a powerful tool that can be used to integrate many types of information, evaluate cost-benefit of interventions, and aid evidence-based decision-making in well-defined regions [7].

We previously conducted a descriptive study of scorpionism in the state of São Paulo (SP) [8], where we described the epidemiological profiles of accidents and deaths in all municipalities of the state from 2008 to 2018. In a second study, we compared the demographic, environmental, and climatic characteristics of the higher- and lower-risk areas in SP for scorpion envenomation [9]. In the present study, we (i) modeled the spatio-temporal variability of scorpionism across 645 municipalities in SP for the period of 2008–2021 using a Bayesian hierarchical approach and (ii) investigated its relationship with demographic, socioeconomic, environmental, and climatic variables. Our hypothesis is that the incidence of scorpionism in a specific region is greatly influenced by both socio-economic and climatic factors.

## Methods

### Ethics statement

The present study was developed using secondary data provided by the CVE (Secretary of Health of the state of São Paulo). The data form which was anonymized without names or addresses, and scorpion accidents were aggregated by municipality and year. The protocol for the present study was submitted for approval by the institutional ethics review board of the University of São Paulo School of Public Health (COEP FSP/USP, CAAE approval record 10457119.6.0000.5421, protocol number 3408558) and no consent was required because we used anonymized secondary data.

### Study area and data collection

São Paulo is located in the southeastern region of Brazil, has an area of around 248,210 km^2^, a population of around 45 million (21.8% of Brazil’s population), and accounts for 33.5% of the national GDP. The median monthly income per capita in 2021 was around US$ 348, but there is huge variation between municipalities reflecting the inequalities present in the state. Currently, 19.28% of the state is covered with native vegetation remnants [10], which mainly include the Cerrado biome and the Atlantic Forest. *Eucalyptus* plantations, sugar cane, and cattle pastures are the predominant agricultural land uses, accounting for approximately 39% of the state’s area [11]. Regarding the climate, São Paulo has three major climate zones. The Koppen-Ginger classification categorizes the western plateau as having a tropical climate (Aw), marked by wet summers and dry winters. The high-altitude regions of the Atlantic plateau and basaltic cuestas have a high-altitude tropical climate (Cwa and Cwb), featuring hot summers and cold winters. The low-lying coastal area has a humid tropical climate (Af), with hot and humid conditions throughout the year. Rainfall varies, with an average of 1,600 mm on the south coast and 2,700 mm on the north coast.

**Fig 1:**
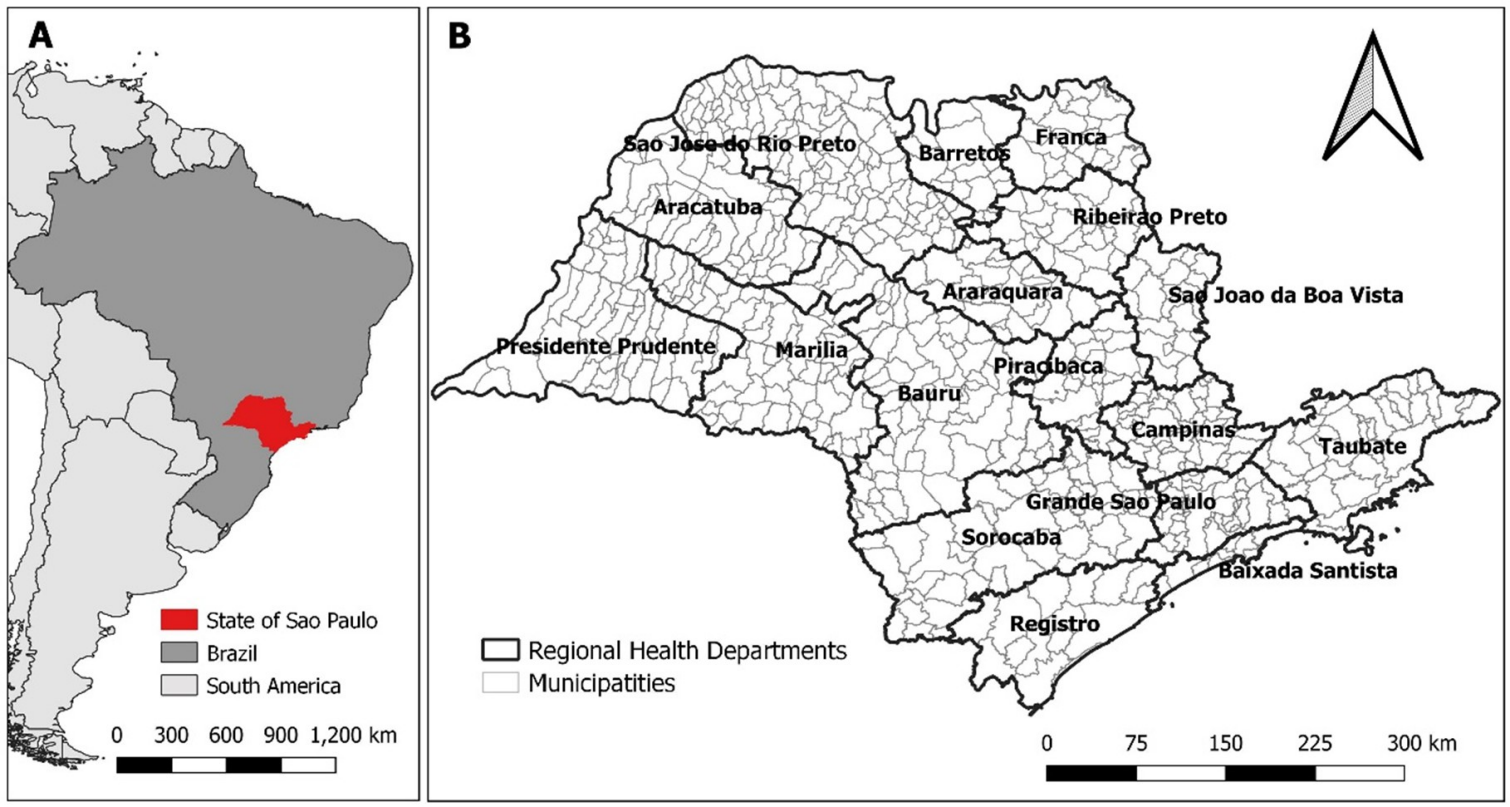
Study area. (A) State of São Paulo in Brazil and South America and (B) municipalities and Regional Health Departments (RHDs) of the state of São Paulo. Base layer of map: https://www.ibge.gov.br/geociencias/organizacao-do-territorio/malhas-territoriais/15774-malhas.html?=&t=acesso-ao-produto.

We analyzed data on scorpionism reported between 2008 and 2021 in 645 municipalities of SP which are aggregated into 17 Regional Health Departments (RHDs) (Fig 1). Data on confirmed cases of scorpion envenomation were obtained from notification forms in the Notifiable Diseases Information System (SINAN) of each municipality, available from the Centre for Epidemiological Surveillance of SP. The data were analyzed in aggregated form (by municipality), so the confidential and specific information of each patient was not accessed, and the consent to data processing was not needed.

**Fig 2:**
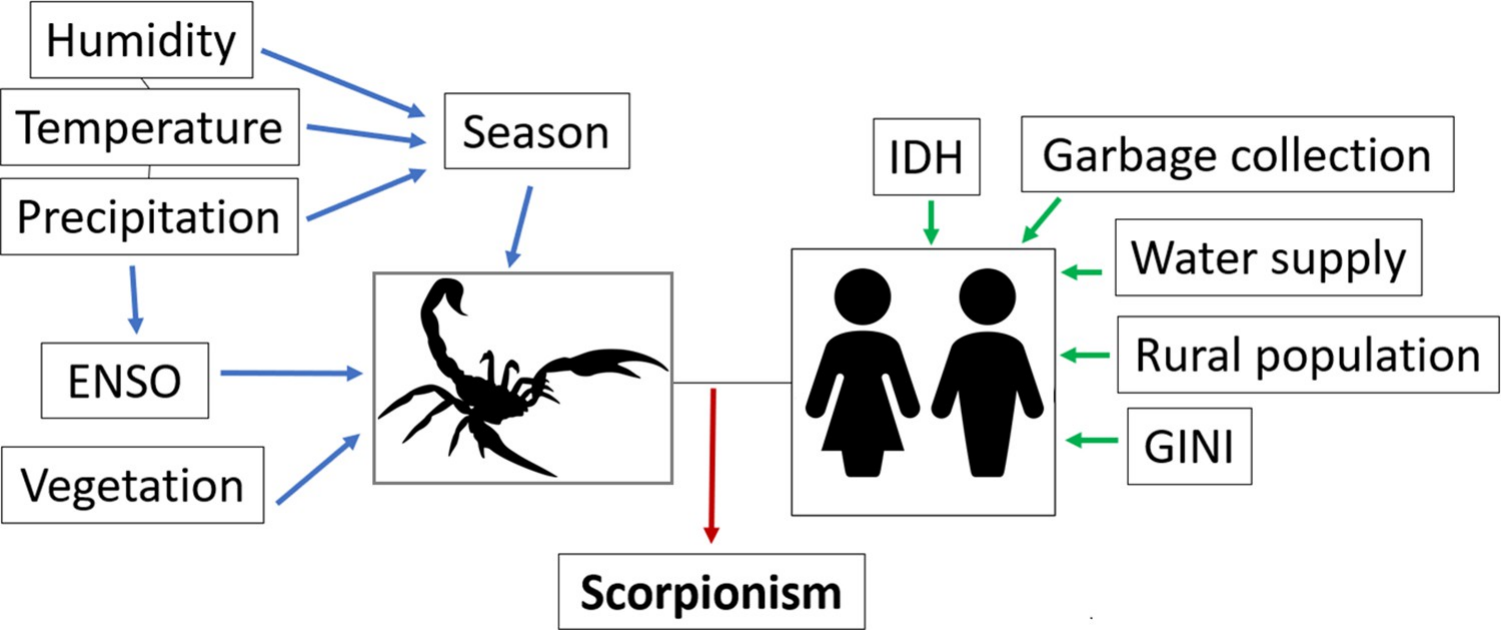
Conceptual framework of scorpionism. We used variables related to human populations (green arrow) and scorpion populations (blue arrow). Not all were included in the statistical models due to collinearity. See the text for details. Source of icons: https://openclipart.org/.

Geographic and demographic information on the municipalities were obtained from the Brazilian Institute of Geography and Statistics (IBGE), including the shape files of the study regions. In terms of environmental variables, we chose those most related to the scorpion life cycle according to previous literature [12,13]: total precipitation (mm), percentage of natural vegetation [14], maximum temperatures (°C), relative humidity (%), and intensity of El Niño-Southern Oscillation (ENSO). We also considered seasonal effects for each year, as reported by previous studies [8,9]. Since there are specific population groups that are more vulnerable to scorpionism, especially those living in poorer areas [15], we also included the following socioeconomic indicators (Fig 2): the Gini index [16], the Human Development Index (HDI) [17], the percentage of rural population, water supply, and garbage collection. The Gini coefficient captures income inequality, and HDI is a composite index that includes three elements: standard of living, life expectancy, and literacy level. All municipality-scale socioeconomic covariates were obtained from the IBGE [18]; the latest available data are for 2010 (last census), and will be used in this analysis to represent of the entire period under study. Environmental and climatic variables were obtained for each season (4) and year (14) combination from the DAEE [19], ESALQ-USP [20], CIIAGRO [21] and IAC [22]. In this study we defined the seasons as follows: "summer" in the months of January- March; "autumn" in the months of April—June; "winter" in July-September; and "spring" in October—December. To access the variables for each season of the year, we aggregated maximum temperatures based on hourly reports, and considered the average of relative humidity.

### Data analysis

The observed data on scorpion envenomations were aggregated by sex and age for each season in each year and municipality. Then, using indirect standardization and the entire study region over the whole period as a reference population, we obtained the expected values after adjusting for age and sex (S1 Appendix). The ratio of observed to expected values represents the standardized morbidity ratio (SMR) for each municipality, season, and year. An exploratory analysis was performed to identify outliers, evaluate the relationship between the response variable (log of SMR) and covariates, and assess potential collinearity among covariates. To do so, we calculated the variance inflation factors (VIF) for a multilinear regression. In accordance with Zuur et al. [23], we considered a VIF larger than 3.00 to indicate collinearity. All continuous covariates were standardized by subtracting the mean and dividing by the standard deviation.

Our response variable was the observed number of accidents in each municipality, season, and year, which we considered to be Poisson distributed. We included the log of the expected values in these models as an offset; therefore, the estimates were expressed as log relative risks (RR). On the log RR, we first specified a disease mapping model to estimate the spatiotemporal variability in scorpion envenomations for the 645 municipalities and each season over the 14 years considered. The model included a global intercept and spatial, temporal, and spatiotemporal random effects. As a second step, we ran an ecological regression model, including covariates, while retaining the random effects. The models were framed from a Bayesian perspective, and statistical inference was performed using the Integrated Nested Laplace Approximation approach (INLA) [24]. The priors for fixed effects (regression coefficients and global intercept) were specified as Gaussian, centered on 0, and with a large variance (minimally informative). The spatial random effects were modeled flexibly to allow for local dependency (i.e., municipalities sharing borders were more likely to be similar) as well as global similarities (i.e., all the municipalities were considered similar as part of the study region) letting the data inform about the relative weight of the two [25]. We used the Queen contiguity weight matrix to represent the local dependency among the municipalities. Similarly, on the temporal random effects, we specified the combination of (i) a structured random effect modeled as random walk of order one (RW1), two (RW2), or autoregressive of order one (AR1), and (ii) an unstructured random effect assuming similarities across all time points in the study. For comparison, we additionally evaluated a parametric trend for the temporal component of the models [26]. Finally, we considered an unstructured spatio-temporal interaction, assuming similarity in space and time after accounting for the main spatial and temporal effects (type I interaction effect) [27]. The priors on the precision of the random effects were specified following Simpson et al. [28], who suggested penalizing model complexity. In particular, they penalized departure from the base model (assuming a constant RR over all areas or time points, i.e., no spatial or temporal variation). We considered all the covariate effects to be linear, except for precipitation and relative humidity, on which we assumed nonlinear relationships with the response variable through RW1 and RW2. As the relationship between temperature and the response variable was less clear, we considered both linear and nonlinear effects (through RW1 and RW2 models). We calculated the Watanabe Akaike Information Criteria (WAIC) for models comparison, choosing the models with the lowest WAIC values [29]. The detailed mathematical specification of the Bayesian hierarchical models is presented in the S1—Mathematical specification of the Bayesian hierarchical models. We ran our models using the R program version 4.2.1 [30] and R-INLA version 22.05.07 [31]. We made the database (S1 Database), the map of the municipalities of state of São Paulo (S1 Map) and the final code (S1 Code) of our modelling approach available for the readers.

## Results

The incidence rate of scorpion envenomation in SP was 30.8 cases per 100,000 inhabitant-years over the entire period from 2008 to 2021, corresponding to more than 250 thousand notifications in SINAN. Among the covariates, there were outliers in the percentages of natural vegetation and rural population, and these were transformed using the square root. Maximum temperature, total precipitation, and relative humidity did not exhibit a linear relationship with the response variable. Using VIF and considering all covariates, we obtained values for total precipitation, relative humidity, and seasonal effects above 3.00, which is the suggested threshold to check for collinearity. While removing relative humidity did not improve the VIF, we found that removing total precipitation resulted in a VIF value below 3; therefore, we used this model specification.

First, we ran the disease-mapping model, and the one with the lowest WAIC is the model with RW1 as the structured temporal random effect (S1 Table). We then compared the following ecological regression models: (i) including all covariates and assuming that relative humidity has a nonlinear relationship with the response variable; (ii) including all covariates and assuming that relative humidity and maximum temperature have a nonlinear relationship with the response variable. The model that presented the lowest WAIC (our final ecological regression) was the one with the maximum temperature and relative humidity modeled as nonlinear through a RW1, and RW2 as the structured temporal random effect (S1 Table).

**Fig 3:**
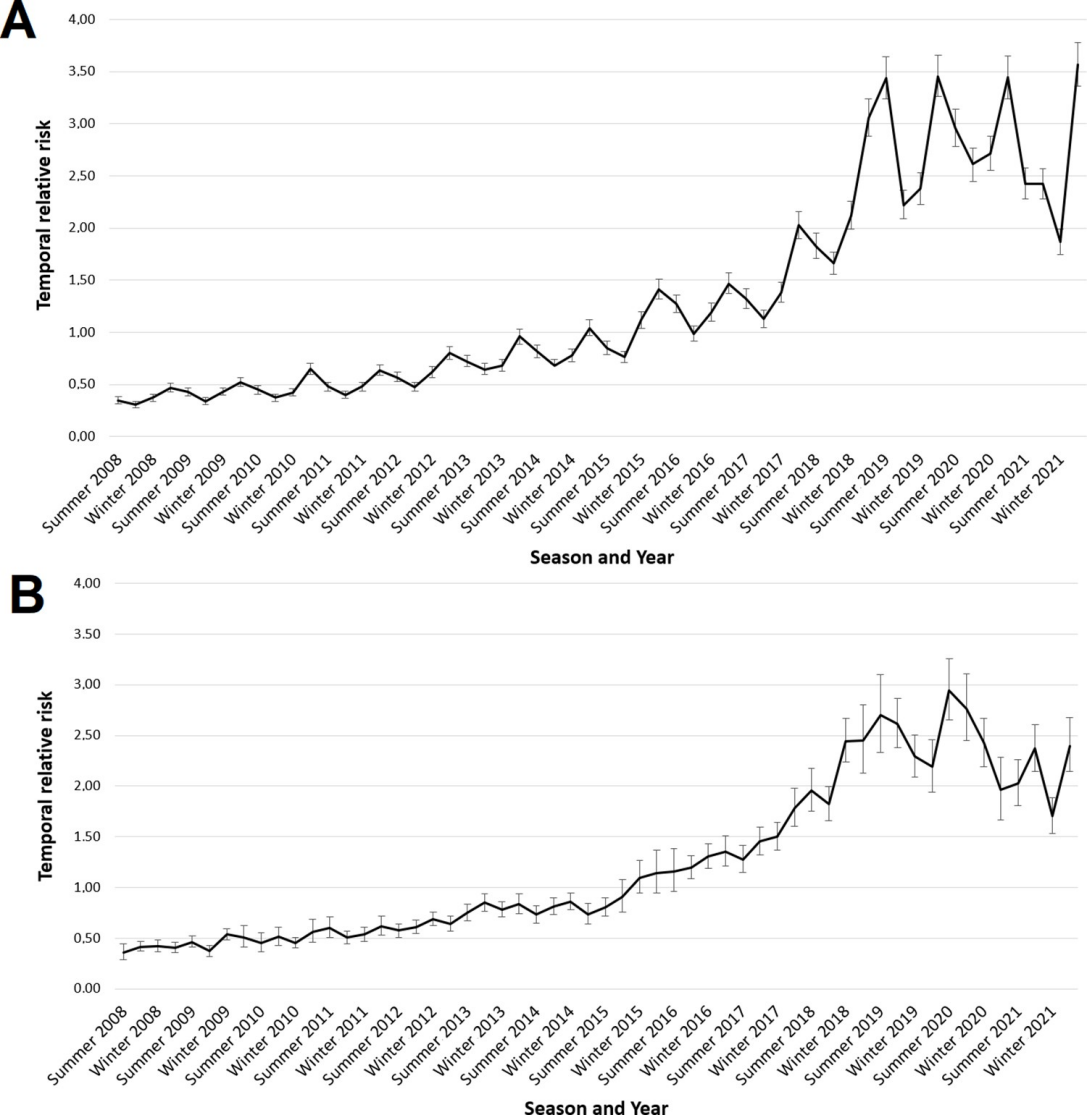
Temporal relative risks. Posterior means and 95% credible intervals of the temporal relative risks (natural scale) obtained from the temporal random effects of (A) the disease mapping model and (B) of the full model for the occurrence of scorpion envenomation in the state of São Paulo by season from 2008 to 2021.

The curve of the disease mapping model shows that the relative risk of occurrence of scorpion envenomation increased continually from 2008 to 2021 in SP, rising from 0.47 (95%CI 0.43–0.51) to 3.57 (95%CI 3.36–3.78) in natural scale; in general, higher values were seen in spring and lower values (except in 2021) in autumn (Fig 3). The temporal curve of the final ecological regression model presented lower values and a larger 95%CI than the curve of the disease-mapping model. This shows that part of the temporal variability in scorpionism was explained by the considered covariates.

**Fig 4:**
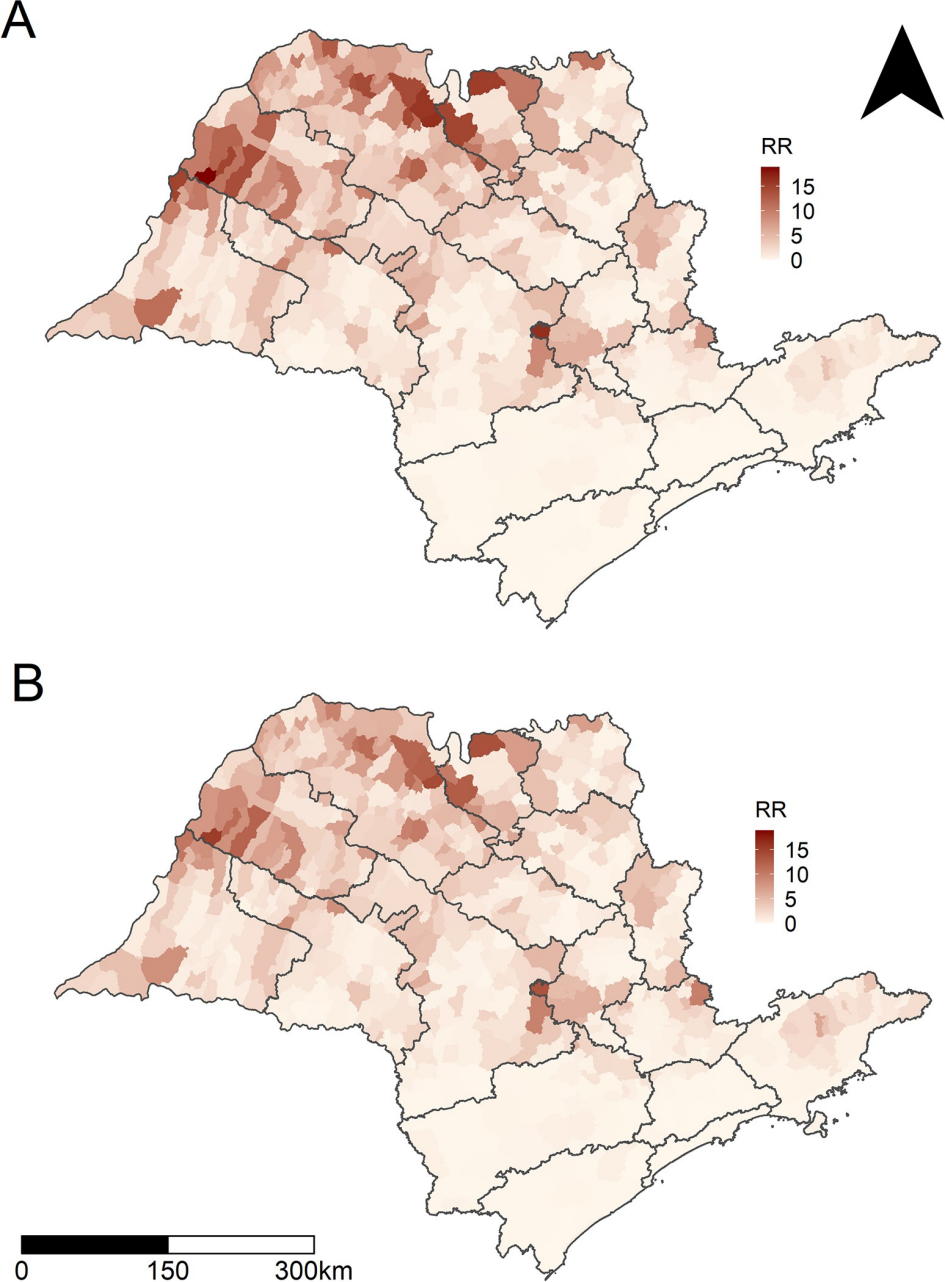
Spatial relative risks. Posterior means of the spatial relative risks (natural scale) obtained from the spatial random effects of (A) the disease mapping model and (B) the final full model for the occurrence of scorpion envenomation in the municipalities of São Paulo, 2008–2021. The municipalities are grouped by Regional Health Departments (see Fig 1). Base layer of map: https://www.ibge.gov.br/geociencias/organizacao-do-territorio/malhas-territoriais/15774-malhas.html?=&t=acesso-ao-produto.

Considering the disease-mapping model and the entire period, the values showed large spatial variability across the municipalities of SP, ranging from 0.0 to 18.9, with some presenting the risk of occurrence of scorpionism almost 19 times the average value for the entire period and state (Fig 4A). Municipalities with higher risks were located in the western, northern, and northwestern parts of SP. The most affected RHDs were Araçatuba, Barretos, Franca, Presidente Prudente, and São José do Rio Preto (Figs 1 and 4).

**Fig 5:**
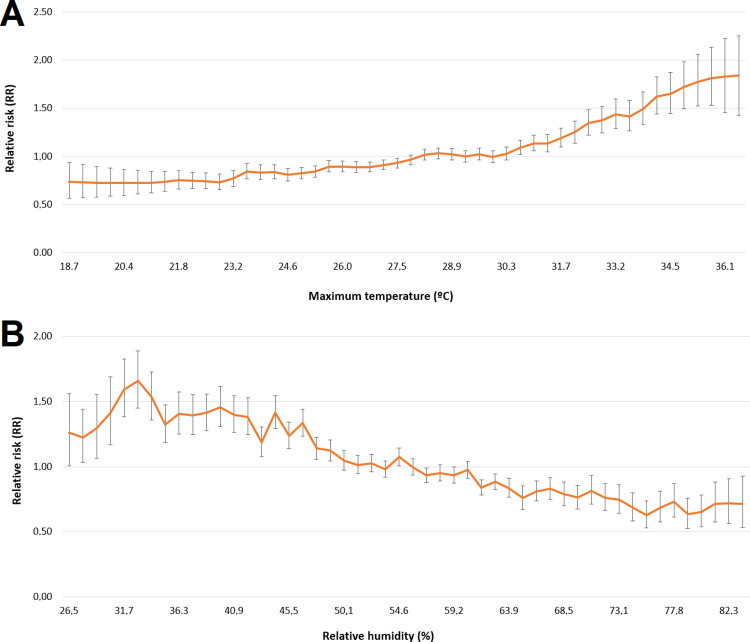
Relationship of scorpionism with environmental variables. Posterior relative risks (natural scale) representing the relationship among the (A) maximum temperature and (B) relative humidity with the scorpion accidents, municipalities of state of São Paulo, Brazil, 2008 to 2021.

Our ecological regression model suggests that the spring and summer seasons are risk factors, showing a 31% and 23% increase in scorpionism risk compared to autumn, respectively (Table 1). In winter, we saw a decrease of 13% in scorpion envenomations. On the other hand, natural vegetation and ENSO showed no significant association with accidents. Higher temperatures and lower humidity were associated with a higher risk of accidents (Fig 5). In particular, for temperatures between 18 and 27°C, the RR ranged between 0.73 (95%CI 0.56–0.94) and 0.93 (95%CI 0.88–0.98), respectively. For higher temperatures, from 34 to 37°C, RR ranged from 1.09 (95%CI 1.02–1.16) to 1.84 (95%CI 1.42–2.25), respectively. Relative humidity increasing from 27 to 33% corresponded to an increase in accident risks ranging from 1.26 (95%CI 1.01–1.56) to 1.66 (95%CI 1.45–1.89). However, for relative humidity between 34 and 84%, the RR decreased steadily from 1.54 (95%CI 1.36–1.73) to 0.71 (95%CI 0.54–0.93). S1 and S2 Figs show relative humidity and maximum temperatures in each municipality of the state of São Paulo, respectively.

**Table 1:** Final full ecological regression models. Posterior means and 95% credible intervals (95% CI) of the relative risks (RR) for the covariates (in natural scale) in the disease mapping and final full ecological regression models for the occurrence of scorpion envenomation, municipalities of the state of São Paulo, 2008 to 2021. The cells in bold indicate that the 95% CI do not include the null risk of one.

Intercept/Covariates		RR	95% CI
**Disease mapping model + random effects**		0.73	0.69–0.76
**Full model**			
Intercept		0.68	0.62–0.75
Seasons	Autumn	1.00	-
	Winter	**0.87**	**0.78–0.97**
	Summer	**1.23**	**1.09–1.39**
	Spring	**1.31**	**1.16–1.48**
ENSO–El Niño Southern Oscillation	Neutral	1.00	-
	El Niño strong	1.06	0.83–1.33
	El Niño moderate	0.89	0.75–1.05
	El Niño weak	0.95	0.85–1.06
	La Niña weak	1.14	0.99–1.30
	La Niña moderate	1.02	0.80–1.28
	La Niña strong	1.14	0.94–1.36
Square root of the natural vegetation		0.96	0.88–1.06
Square root of the rural population		0.98	0.91–1.06
Municipal human development index		0.95	0.87–1.04
Gini index		**1.11**	**1.01–1.23**
Percentage of population with water supply		1.03	0.94–1.13
Percentage of population with garbage collection		1.06	0.98–1.14

The Gini index was the only socioeconomic variable that showed a positive association with the response variable, with an increase of one standard deviation corresponding to a 11% increase in accident risk. The purpose of the Gini index is to demonstrate the distribution of income within an economy (S3 Fig). In other words, a higher value of the Gini index indicates a higher degree of economic inequality in a given area. Other factors considered in our model such as rural population, HDI, water supply, and garbage collection, showed no significant association with accidents (Table 1).

**Fig 6:**
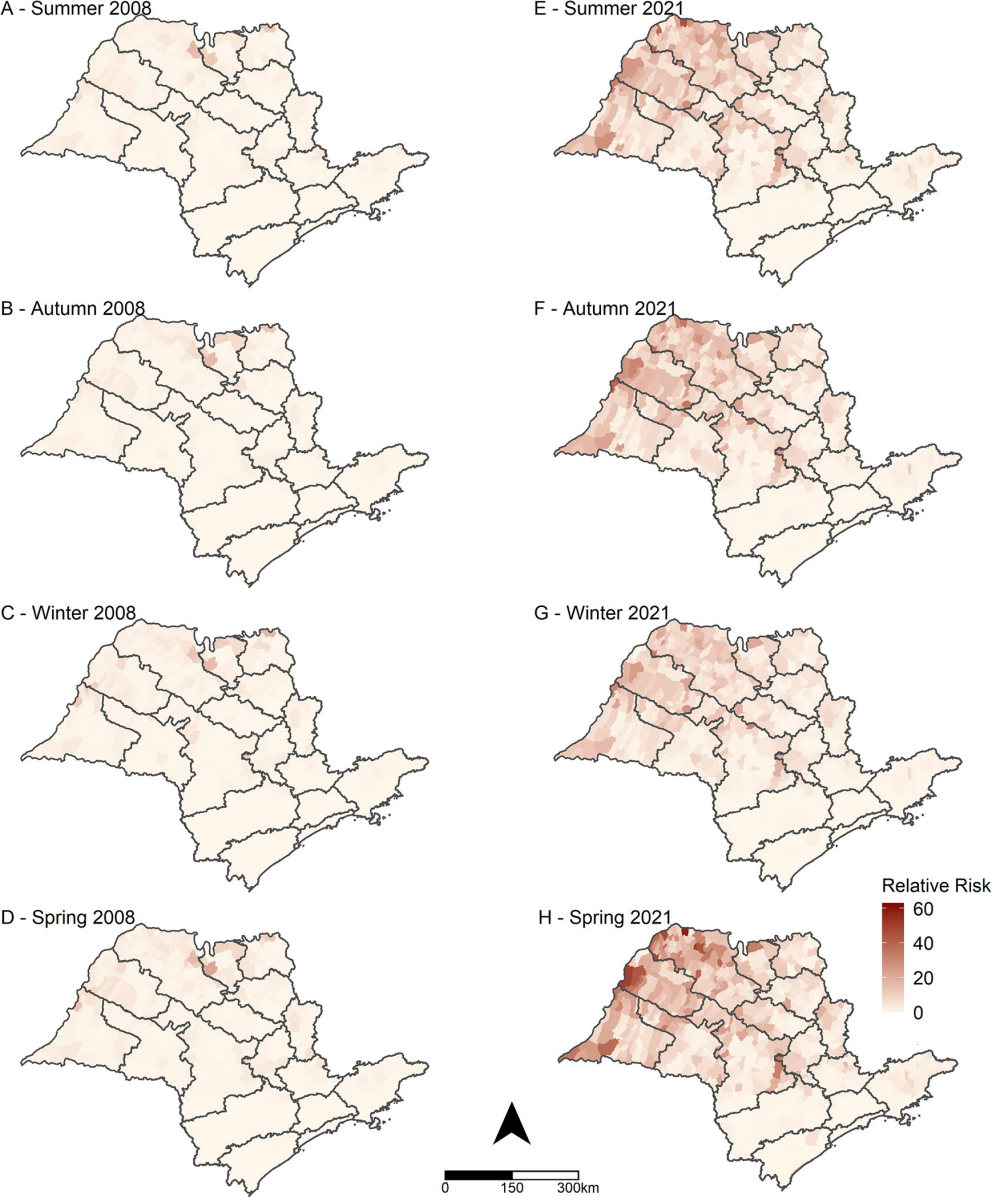
Predicted spatial relative risks. Posterior means of the predicted relative risks in natural scale obtained from the ecological regression model, for the four seasons of 2008 and 2021, municipalities of the state of São Paulo. The municipalities are grouped by Regional Health Departments (see Fig 1). Base layer of map: https://www.ibge.gov.br/geociencias/organizacao-do-territorio/malhas-territoriais/15774-malhas.html?=&t=acesso-ao-produto.

**Fig 7:**
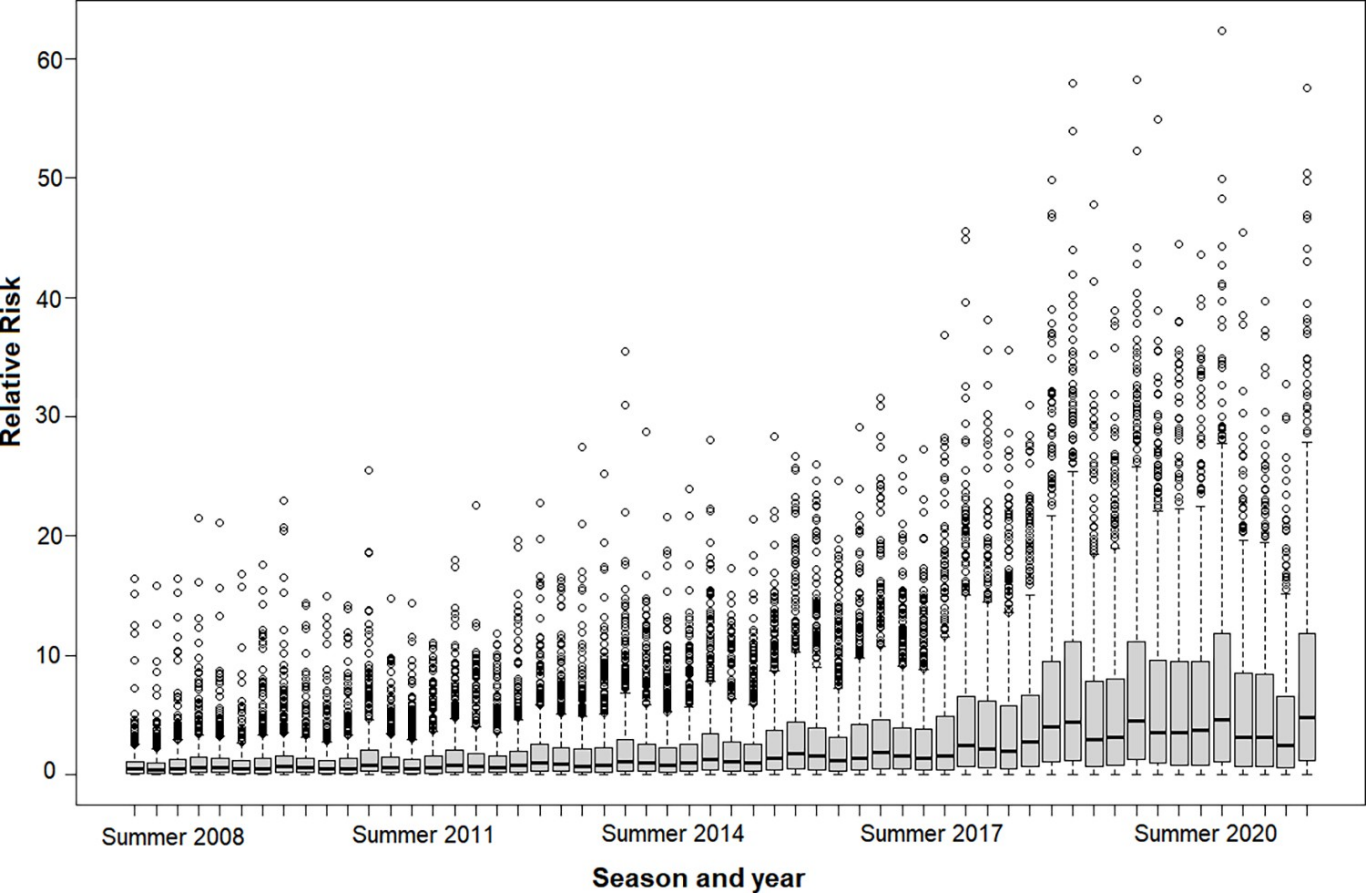
Predicted temporal relative risks. Posterior means of the predicted relative risk in natural scale of the full ecological regression model for the municipalities of the state of São Paulo presented in boxplot for each season and year from 2008 to 2021.

Our data revealed a significant increase in the risk of scorpion accidents between 2008 and 2021, with spring being the season with the highest risk, and the western, northwestern, and northern regions of SP being the most affected areas (Fig 6). Regarding the predicted RR for the full model considering each municipality, we showed that the risk for scorpionism increased in almost all municipalities of SP from 2008 to 2021 (Fig 7).

## Discussion

SINAN reported a progressive increase in the number of notifications related to venomous animals each year in Brazil [6,32]. Our analysis revealed an upward trend in the number of scorpion accidents in SP: from the spring of 2008 to 2021, the RR increased eight times. This growth trend is corroborated by studies in other states of the Brazilian southeast [33] and northeast regions [34,35]. It is unknown how much of the increase can be attributed to improvements in the notification system, but also reveals the worrisome affinity of many scorpion species for anthropically modified environments, which consequently enhanced their contact with humans [2,36]. Unplanned urban growth, indiscriminate use of natural resources, industrialization, and ecological imbalance induce the spread of some venomous species and increase the overlap between the areas used by these animals and humans [35,37]. Amado et al. [36] confirmed the affinity of *T*. *serrulatus* for altered environments, as its modeled distribution was highly correlated with human population density in Brazil. The preference of this species for anthropically modified environments appears to be related to its parthenogenetic reproduction, as this type of reproduction is frequent in uniform environments such as urban areas [12]. The growth of cities can also be considered as another determining factor in the proliferation of scorpions, as the generation and accumulation of debris play a key role in the availability of breeding habitats, as they facilitate the spread of cockroaches and other insects that are food sources.

Our results showed that RR increased continually from 2008 to 2018; however, since 2019, there has been an apparent stabilization in the number of cases. One hypothesis that can be raised is the underreporting of cases due to the COVID-19 pandemic, especially in 2020 and 2021 [38]. The efforts of epidemiological surveillance teams in several municipalities likely concentrated on facing the COVID-19 pandemic and this may have been reflected in the scorpion survey. Moreover, as people spent more time at home, they took more time to care and clean their backyards, reducing ideal habitats for scorpions.

Our findings showed that the spring and summer seasons were the periods with the highest risks, showing, respectively, 26% and 19% increase in scorpion accident risks compared to autumn, while winter showed a decrease of 13% in accident risks. The state of SP is located in a tropical area, and the four seasons of the year are not well defined. Nevertheless, spring tends to exhibit high temperatures. Related findings have been reported in other Brazilian regions [39,40] and other countries [41–44] where scorpion envenomation have been more recurrent during the hotter seasons of the year. However, some studies [1] indicate constant scorpion incidents over all months of the year, which could be explained by ideal climatic conditions and abundant food throughout the year [1,9]. The results from our model suggest that maximum temperature is a risk factor, whereas relative humidity is a protective factor against the occurrence of scorpion accidents. Comparing Figs 4 and 6 with S1 and S2 Figs, it can be seen that the regions with higher scorpion accident rates are the same regions with higher maximum temperatures and lower relative humidity. Scorpions are animals that are extremely adaptable to extreme environmental conditions [45], and they are particularly abundant in desert areas [46].

Municipalities with higher risk were located in the western, northern, and northwestern parts of SP. The most affected RHDs were Araçatuba, Barretos, Franca, Presidente Prudente, and São José do Rio Preto, as described previously [8,9]. Other studies have also implicated these areas as hotspots of medical concern for scorpionism [47]. All higher-risk regions detected here also exhibited higher temperatures and lower precipitation, corroborating our model. Our findings are also in agreement with those of Moradiasl et al. [48] and Amado et al. [36], who reported that air temperature and precipitation are key variables that influence the distribution of scorpions in Iran and Brazil, respectively. Rafinejad et al. [49] analyzed climatic variations as crucial elements for the presence of scorpions in Iran, as well as their stimulated life cycle and maturation. Many other studies carried out in Brazil reveal that climatic variables play a key role in the distribution of scorpions and, consequently, accidents [50]. Given the increasing effects of climate change and deforestation on Brazilian biomes, it is expected that the scorpion population will grow and lead to a surge in scorpionism, unless measures are taken to prevent it from happening [50]. To address this issue, the implementation of educational programs for the prevention and treatment of scorpion envenomation, aimed at community members and health workers, could be a valuable public health policy to help reduce the rising number of incidents.

In our model, the Gini index showed a positive association with scorpionism since an increase of one standard deviation represented a 13% increase in accident risk. The higher the Gini coefficient, the greater the income inequality, and consequently, the greater the differences in housing conditions and access to healthcare. Poor housing conditions and a lack of basic sanitation were found to be associated with an increase in scorpionism in Sergipe, Brazil [51]. The association between areas with many accidents and those with high vulnerability has also been reported by Almeida et al. [15]. A higher incidence of scorpion stings usually appears in regions of older and crowded favelas, characterized by inefficient basic sanitation and other substandard housing conditions [52]. Many municipalities located in the western, northern, and northwestern parts of the SP experienced a verticalization process and increased urbanization which has led to more suitable anthropogenic habitat for scorpions.

The other variables investigated (including ENSO, natural vegetation, rural population, water supply, garbage collection, and HDI) did not show evidence of an association with accident risks. However, caution is needed when interpreting these results, as this is an ecological study, and an ecological fallacy could have been induced. Commonly, the “ecological fallacy” refers to the error of assuming that statistical relationships at a group level also hold for individuals in the group [53]. However, it is increasingly recognized that population-level research plays a crucial role in characterizing the most important public health issues to be tackled and in developing hypotheses regarding their probable causes. Future studies of the individual risk for scorpionism, such as cohort or case-control approaches, should be carried out to confirm whether the variables analyzed here are actual risk factors.

Regarding the limitations of this study, we highlight the use of secondary data, which has already been collected and recorded by health facilities. A possible bias could be associated with this issue; the notification system is susceptible to underreporting, which can show incidences lower than real occurrence numbers. Nevertheless, the system has been enhanced over time, which is reflected in both the increase in notifications and in the requirements for health care [2]. Despite these limitations, the SINAN database of the Brazilian Ministry of Health was considered to be an essential tool for conducting several epidemiological studies, given that it is the official system for reporting diseases and health accidents in Brazil [1]. In addition, the socioeconomic variables used in our analysis are outdated; to date, there is only information from the last census carried out by the IBGE in 2010. Another limitation is that we did not consider data regarding the infestation and abundance of scorpion species in SP. However, this is the first study to evaluate the relationship with potential risk factors while accounting for spatiotemporal dependencies over such a long period. In addition, spatiotemporal data naturally accounts for residual confounding [54]. Our study offers a methodology for investigation that can provide indications of geographical areas and seasonal periods that are at higher risk for accidents, thereby generating insights for the development of effective intervention strategies, which should be locally and temporally targeted.

The Brazilian Ministry of Health [4] suggests taking preventive measures such as keeping households and surrounding areas clean and free of piled up waste, sealing doors, windows, drains, and walls to prevent scorpions from entering homes, checking shoes and clothing before putting them on, removing potential scorpion prey like cockroaches, and attracting natural scorpion predators like birds, lizards, and frogs [50]. Effective control of scorpions, similar to the control of mosquito-borne diseases, requires active participation and engagement from communities. Both scorpions and mosquitoes are urban pests that affect households, and lessons can be learned from existing vector control strategies. However, engaging communities is a complex process that goes beyond simply sharing information. It is essential to involve stakeholders in decision making and actions, and to have a deep understanding of the socio-political, economic, and cultural context of the communities to be able to establish meaningful dialogue and develop successful plans that foster community involvement [50].

Our results indicate that reducing the negative effects of scorpion envenomation requires a collaborative effort from multiple teams, such as environmental management, healthcare workers, researchers, and the general public. Destoumieux-Garzón et al. [55] have stated that the concepts of human health, public health, sanitation, environmental health, and animal health are interrelated. This accumulated knowledge gave rise to the term "one health," recognizing that human activities and factors connected to socioeconomic and environmental circumstances can worsen the occurrences of diseases, fatalities, and injuries, particularly among vulnerable populations. Therefore, further in-depth studies that focus on individuals are necessary to gain a better understanding of the epidemiology of scorpion incidents, and this information can be used to develop educational healthcare measures to enhance the support provided to those who have been affected.

## Conclusion

This study showed that the RR of scorpion accidents in the state of São Paulo increased eight-fold between 2008 and 2021. Municipalities with higher risk were in the western, northern, and northwestern parts of the SP. The most affected RHDs were Araçatuba, Barretos, Franca, Presidente Prudente and São José do Rio Preto. Accidents occurred more frequently in spring, while winter showed a protective effect, with a decrease of 13% in accident risk. In our model, the Gini index, which captures income inequality, shows a positive association with scorpionism. Other variables such as higher temperatures and lower humidity were also associated with a higher risk of accidents in the municipalities, corroborating what has been found in other surveys in Brazil and other countries. Future studies based on individual scorpionism, such as cohort or case-control approaches, should be conducted to confirm whether the variables analyzed here are actual risk factors. Additionally, future studies also should address issues related to the process of land occupation as well as the economic functions performed by municipalities and relate them to socio-environmental determinants.

## Supporting information

S1 Appendix Mathematical specification of the Bayesian hierarchical model. (DOCX)

S1 Database Database on confirmed cases of scorpion envenomation from 2008 to 2021 obtained from notification forms in the Notifiable Diseases Information System (SINAN), available from the Centre for Epidemiological Surveillance of SP. (XLSX)

S1 Map Map of the municipalities of the state of São Paulo. Please open with some Geographic Information System software.
(ZIP)

S1 Code Final code of our modelling approach used in R software. (R)

S1 Table WAIC values of the disease mapping model and two possible full ecological regression models. We considered three types of structured temporal random effects—RW1, RW2, and AR1, and two types of non-linear effects for the climatic covariates: RW1 and RW2.
(DOCX)

S1 Fig Maximum temperatures. Maximum temperatures of the municipalities of the state of Sao Paulo by seasons, for 2008 and 2021. Base layer of map: https://www.ibge.gov.br/geociencias/organizacao-do-territorio/malhas-territoriais/15774-malhas.html?=&t=acesso-ao-produto.
(PNG)

S2 Fig Relative humidity. Relative humidity of the municipalities of the state of Sao Paulo by seasons, for 2008 and 2021. Base layer of map: https://www.ibge.gov.br/geociencias/organizacao-do-territorio/malhas-territoriais/15774-malhas.html?=&t=acesso-ao-produto.
(PNG)

S3 Fig Gini index. Gini index of the municipalities of the state of Sao Paulo, 2010. Base layer of map: https://www.ibge.gov.br/geociencias/organizacao-do-territorio/malhas-territoriais/15774-malhas.html?=&t=acesso-ao-produto.
(PNG)
