# Supplementary material for: Spatiotemporal bayesian modelling of scorpionism and its risk factors in the state of São Paulo, Brazil
Source: PLoS Negl Trop Dis. 2023 Jun 20;17(6):e0011435. doi: 10.1371/journal.pntd.0011435 (PMC10313024; doi:10.1371/journal.pntd.0011435)
Supplement: S1 Appendix — (DOCX) [file pntd.0011435.s001.docx]

**S1 - Mathematical specification of the Bayesian hierarchical models**

Let $y_{it}$ be the number of scorpion accidents in $i=1,2,\ldots,645$ municipalities and $t=1,2,\ldots, 56$season-years (i.e., four seasons in 14 years). Additionally, let $E_{it}$ be the expected number of scorpion accidents in municipality $i$ and season-year $t$*,* computed through indirect standardization adjusting for age and sex, that is considering the scorpion accident rates over the entire study region by age and sex, based on the numbers of inhabitants by age and sex in each municipality, season and year (in particular, we considered the following groups for age: 0 to 9, 10 to 19, 20 to 39, 40 to 59, and 60 and more years). We modelled the number of accidents $y_{it}$ assuming a Poisson distribution and we specified a dynamic nonparametric formulation for the linear predictor as follows:

$$y_{it}| \mu_{it}\sim Poisson(\mu_{it})$$

$$\log\left( \mu_{it} \right)=\log\left( E_{it} \right)+\eta_{it}$$

$\eta_{it}=\alpha+\sum_{j=1}^{n} \beta_{j}X_{itj}+\sum_{s=1}^{g} \xi_{s}W_{is}+\theta_{k_{i}}+\psi_{I_{t}}+f_{1}\left( Z_{it1} \right)+f_{2}(Z_{it2})+b_{i}+\gamma_{t}+\omega_{t}+\delta_{it}$ (1)

Here $\eta_{it}$ is the log relative risk (RR) of scorpion accidents in municipality $i$ and season-year $t$. In the model equation, *α* is the global intercept, $\beta_{1},\ldots,\beta_{n}$ are the fixed effects related to the linear covariates $\boldsymbol{X}_{1},\ldots,\boldsymbol{X}_{n}$ varying in space and time, while $\xi_{1},\ldots,\xi_{g}$are the fixed effects of the socioeconomic covariates $\boldsymbol{W}_{1},\ldots,\boldsymbol{W}_{g}$ varying only in space; $\theta_{k_{i}}$is the fixed effect for ENSO as categorical variable ( $k_{i}$= 0: Neutral [reference], $k_{i}$= 1: El Niño strong, $k_{i}$= 2: El Niño moderate, $k_{i}$= 3: El Niño weak, $k_{i}$= 4: La Niña weak, $k_{i}$= 5: La Niña moderate, $k_{i}$= 6: La Niña strong); $\psi_{I_{t}}$ is the indicator for season ($I_{t}$= 0: Autumn [reference], $I_{t}$= 1: Winter, $I_{t}=$2: Summer, $I_{t}$= 3: Spring). Also, $f_{1}$ and $f_{2}$ represent the nonlinear effects of maximum temperature and relative humidity and we considered the following two specifications:

1. nonstationary random walk of order 1 (RW1), which for a generic covariate is specified as: $Z_{it}|Z_{i\left( t-1 \right)}{,\sigma}_{Z}^{2} \sim Normal\left( Z_{i\left( t-1 \right)}, \sigma_{Z}^{2} \right);$

2. nonstationary random walk of order 2 (RW2), which for a generic covariate is specified as: $Z_{it}|Z_{i\left( t-1 \right)}{Z_{i\left( t-2 \right)},\sigma}_{Z}^{2} \sim Normal\left( 2Z_{i\left( t-1 \right)}-Z_{i\left( t-2 \right)}, \sigma_{Z}^{2} \right).$

The spatial field $b_{i}$ is modelled as an extension of the popular Besag-York-Mollié model [1], which takes into account that data may be spatially correlated and is formulated as the sum of an unstructured random effect $v_{i}\sim Normal\left( 0, \sigma_{v}^{2} \right)$ and a spatially structured effect $u_{i}$ specified through an intrinsic conditional autoregressive model. In detail, $b_{i}$ is defined as follows: $b_{i}=\frac{1}{\sqrt{\tau_{b}}}(\sqrt{1-\phi} v_{i}^{*}$ +$\sqrt{\phi}$ $u_{i}^{*})$, where $v_{i}^{*}$ and $u_{i}^{*}$ are standardised versions of $v_{i}$ and $u_{i}$ to have variance equal to one; $\tau_{b}$ is the marginal precision parameter controlling the marginal variance of the random effects and $0\leq\phi\leq1$ is the mixing parameter measuring the proportion of the marginal variance explained by the structured effect [2]. In equation (1), $\gamma_{t}$is a temporally structured component modelled dynamically; we considered different specifications: (i) RW1 (specified as above); (ii) RW2 (specified as above); (iii) a stationary autoregressive process of first order (AR1), specified as $\gamma_{t}\mid\gamma_{t-1},\rho,\sigma_{\gamma}^{2} \sim$ *Normal*(${\rho\gamma}_{t-1}, \sigma_{\gamma}^{2}$), where $|\rho|<1$ is the temporal correlation term. For $\omega_{t}$ we considered an unstructured temporal random effect modelled as $\omega_{t}\sim Normal\left( 0, \sigma_{\omega}^{2} \right)$. Finally, $\delta_{it}$ is an interaction between space and time, which is modelled assuming similarity in space and time after having accounted for the spatial and temporal main effects (i.e., type I space-time interaction [3]) and modelled as $\delta_{it}\sim Normal\left( 0, \sigma_{\delta}^{2} \right)$. For comparison, we additionally evaluated a parametric trend for the temporal component of the model, as proposed by Bernardinelli et al. (1995) [4]. To do so, we replaced the terms $\gamma_{t}$, $\omega_{t}$ and $\delta_{it}$of equation (1), with the following structure $(\gamma+\delta_{i}) T$. In this specific formulation, the parameter $\gamma$ represents the main linear time trend (i.e., the global effect of time$T$) and $\delta_{i}\sim Normal\left( 0, \sigma_{\delta}^{2} \right)$represents the difference between the global trend $\gamma$ and the area-specific trend (i.e., if $\delta_{i}<0$, then the area-specific trend is less steep than the mean trend, whilst $\delta_{i}>0$implies that the area-specific trend is steeper than the mean trend [3]).

We specified minimally informative prior distributions for the fixed effects, while for the hyperparameters of the spatial field $\phi$ and $\tau_{b}$ we used penalized complexity (PC) priors [5], defining the priors using probability statements. In particular, the prior for the marginal precision is defined on the standard deviation and we specified $P\left( \frac{1}{\sqrt{\tau_{b}}}>1 \right)=0.01$. The prior for the mixing parameter $\phi$ is specified as $P\left( \phi<0.5 \right)=0.5$, which reflects the lack of knowledge about which spatial component, the unstructured or structured, accounts for a higher proportion of the variability. We additionally used PC priors for the following standard deviations $\sigma_{v}$, $\sigma_{w}$, $\sigma_{z}$, $\sigma_{\delta}$so that $P\left( \sigma<1 \right)=0.01$.

**References**

1. Besag, J., York, J., Mollié, A. Bayesian image restoration, with two applications in spatial statistics. Ann. Inst. Stat. Math. 1991; 43, 1–20.
2. Riebler, A., Sørbye, S.H., Simpson, D., Rue, H. An intuitive Bayesian spatial model for disease mapping that accounts for scaling. Stat. Methods Med. Res. 2016; 25, 1145–1165.
3. Blangiardo, M., Cameletti, M., Spatial and Spatio-temporal Bayesian Models with R-INLA, 2015, John Wiley & Sons.
4. Bernardinelli, L., Clayton, D., Pascutto, C., Montomoli, C., Ghislandi, M., Songini, M. Bayesian analysis of space–time variation in disease risk. Stat. Med. 1995; 14, 2433–2443.
5. Simpson, D., Rue, H., Riebler, A., Martins, T.G., Sørbye, S.H. Penalising model component complexity: a principled, practical approach to constructing priors. Stat. Sci. 2017; 32, 1–28.
