# Supplementary material for: Spatiotemporal bayesian modelling of scorpionism and its risk factors in the state of São Paulo, Brazil
Source: PLoS Negl Trop Dis. 2023 Jun 20;17(6):e0011435. doi: 10.1371/journal.pntd.0011435 (PMC10313024; doi:10.1371/journal.pntd.0011435)
Supplement: S1 Table — We considered three types of structured temporal random effects—RW1, RW2, and AR1, and two types of non-linear effects for the climatic covariates: RW1 and RW2. (DOCX) [file pntd.0011435.s005.docx]

**S5 – Table - WAIC values of the disease mapping model and two possible full ecological regression models. We considered four types of structured temporal random effects: RW1, RW2, AR1, and parametric trend; and two non-linear effects for the climatic covariates: RW1 and RW2.**

| **Model** | **Covariates** | **Structured temporal random effects** | **Non-linear effects** | **WAIC** |
| --- | --- | --- | --- | --- |
| Disease mapping | No covariates | RW1 | - | **125449.0** |
|  |  | RW2 | - | 125466.1 |
|  |  | AR1 | - | 125476.9 |
|  |  | Parametric Trend | - | 172094.8 |
| Ecological regression | Linear effect for maximum temperature and  nonlinear effect of relative humidity | RW1 | RW1 | **125248.0** |
|  |  |  | RW2 | 125279.6 |
|  |  | RW2 | RW1 | **125248.0** |
|  |  |  | RW2 | 125287.5 |
|  |  | AR1 | RW1 | 125260.4 |
|  |  |  | RW2 | 125278.1 |
|  |  | Parametric trend | RW1 | 160229.1 |
|  |  |  | RW2 | 161186.7 |
|  | Nonlinear effect for maximum temperature and relative humidity | RW1 | RW1 | 125212.2 |
|  |  |  | RW2 | 125291.5 |
|  |  | RW2 | RW1 | **125185.7** |
|  |  |  | RW2 | 125251.8 |
|  |  | AR1 | RW1 | 125232.6 |
|  |  |  | RW2 | 125293.8 |
|  |  | Parametric trend | RW1 | 167146.0 |
|  |  |  | RW2 | 166212.3 |
