# Supplementary figures and images for: Spatiotemporal bayesian modelling of scorpionism and its risk factors in the state of São Paulo, Brazil
Source: PLoS Negl Trop Dis. 2023 Jun 20;17(6):e0011435. doi: 10.1371/journal.pntd.0011435 (PMC10313024; doi:10.1371/journal.pntd.0011435)

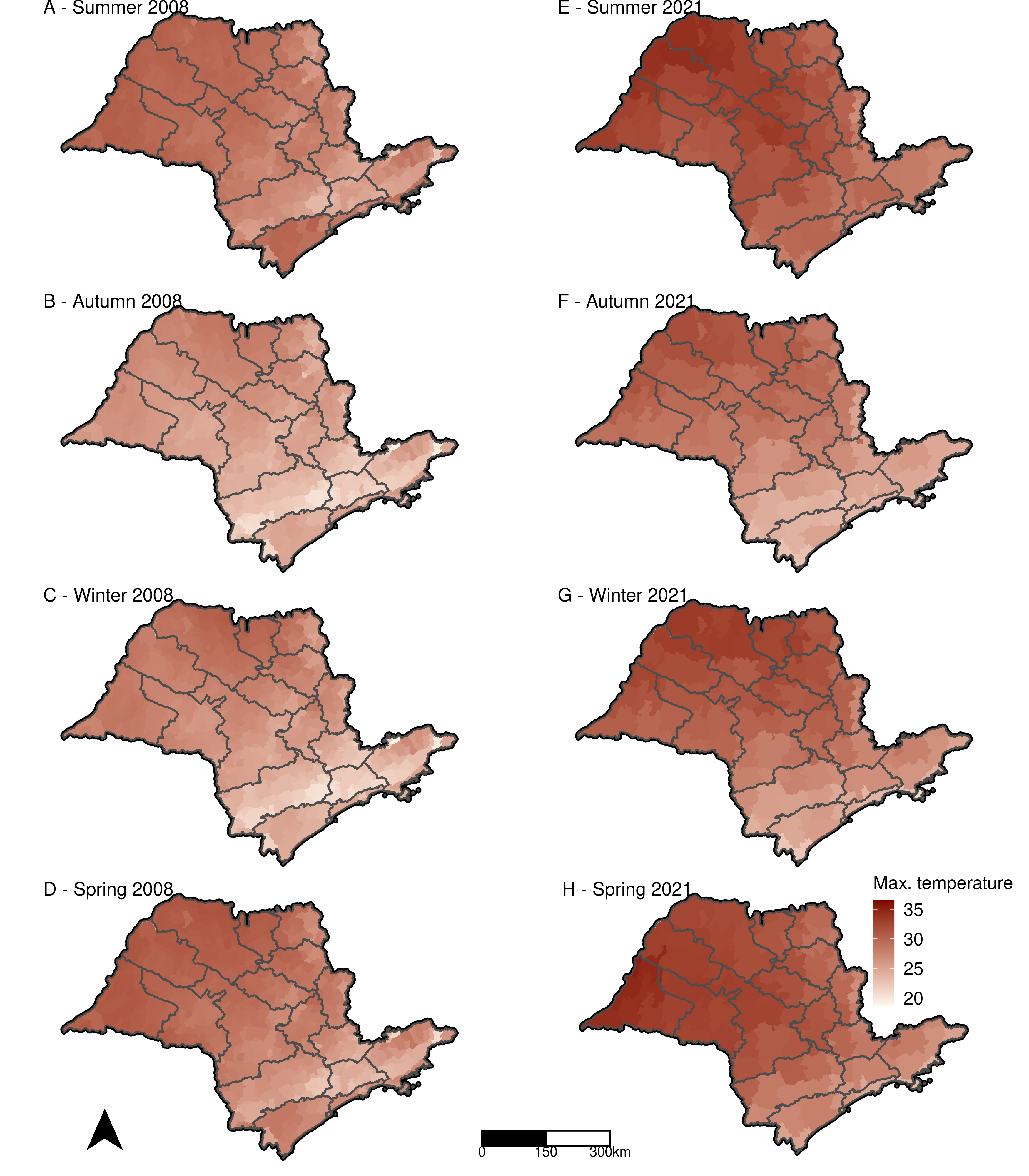

Supplement: S1 Fig — Maximum temperatures of the municipalities of the state of Sao Paulo by seasons, for 2008 and 2021. Base layer of map: https://www.ibge.gov.br/geociencias/organizacao-do-territorio/malhas-territoriais/15774-malhas.html?=&t=acesso-ao-produto. (PNG) [file pntd.0011435.s006.png]

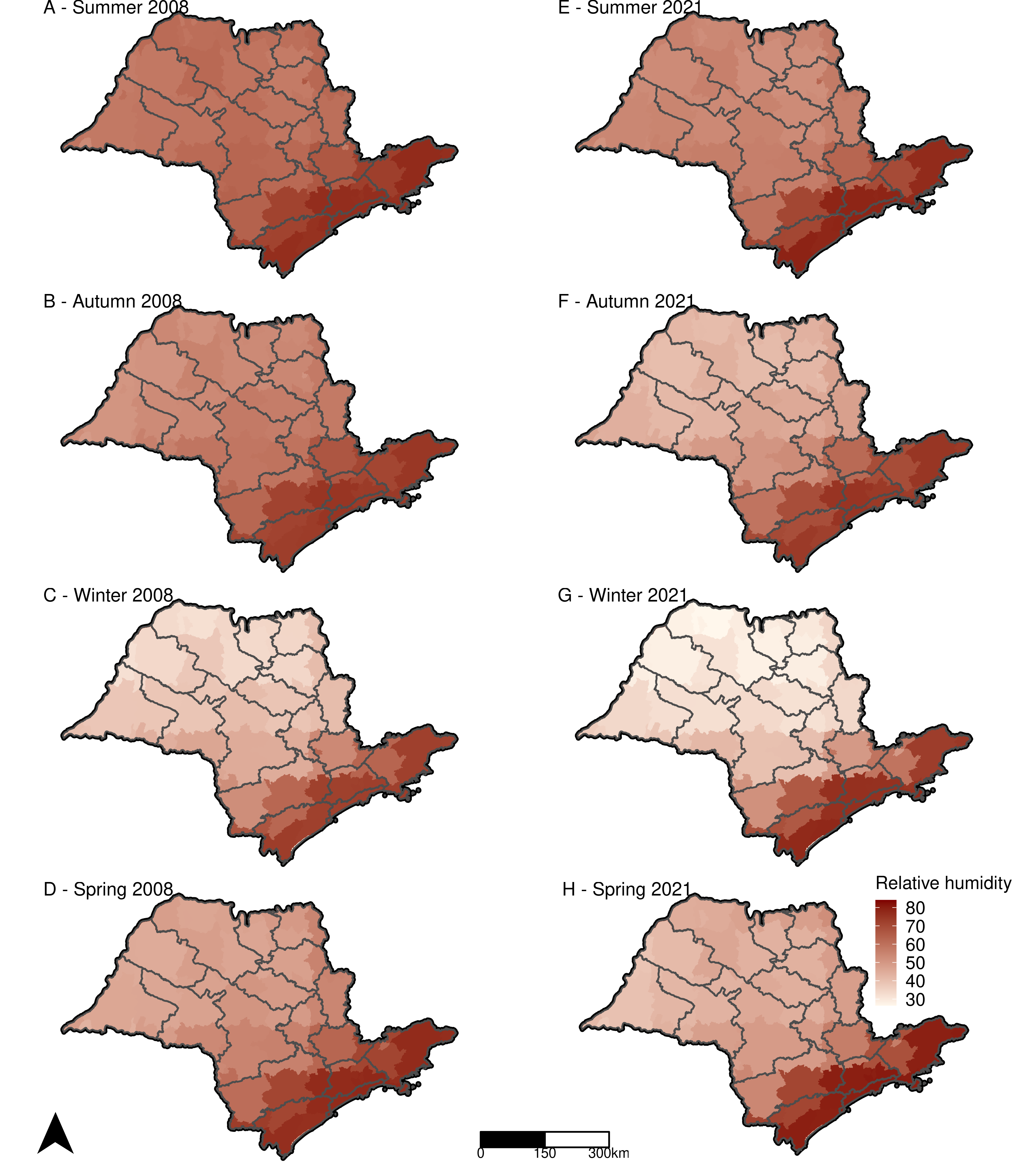

Supplement: S2 Fig — Relative humidity of the municipalities of the state of Sao Paulo by seasons, for 2008 and 2021. Base layer of map: https://www.ibge.gov.br/geociencias/organizacao-do-territorio/malhas-territoriais/15774-malhas.html?=&t=acesso-ao-produto. (PNG) [file pntd.0011435.s007.png]

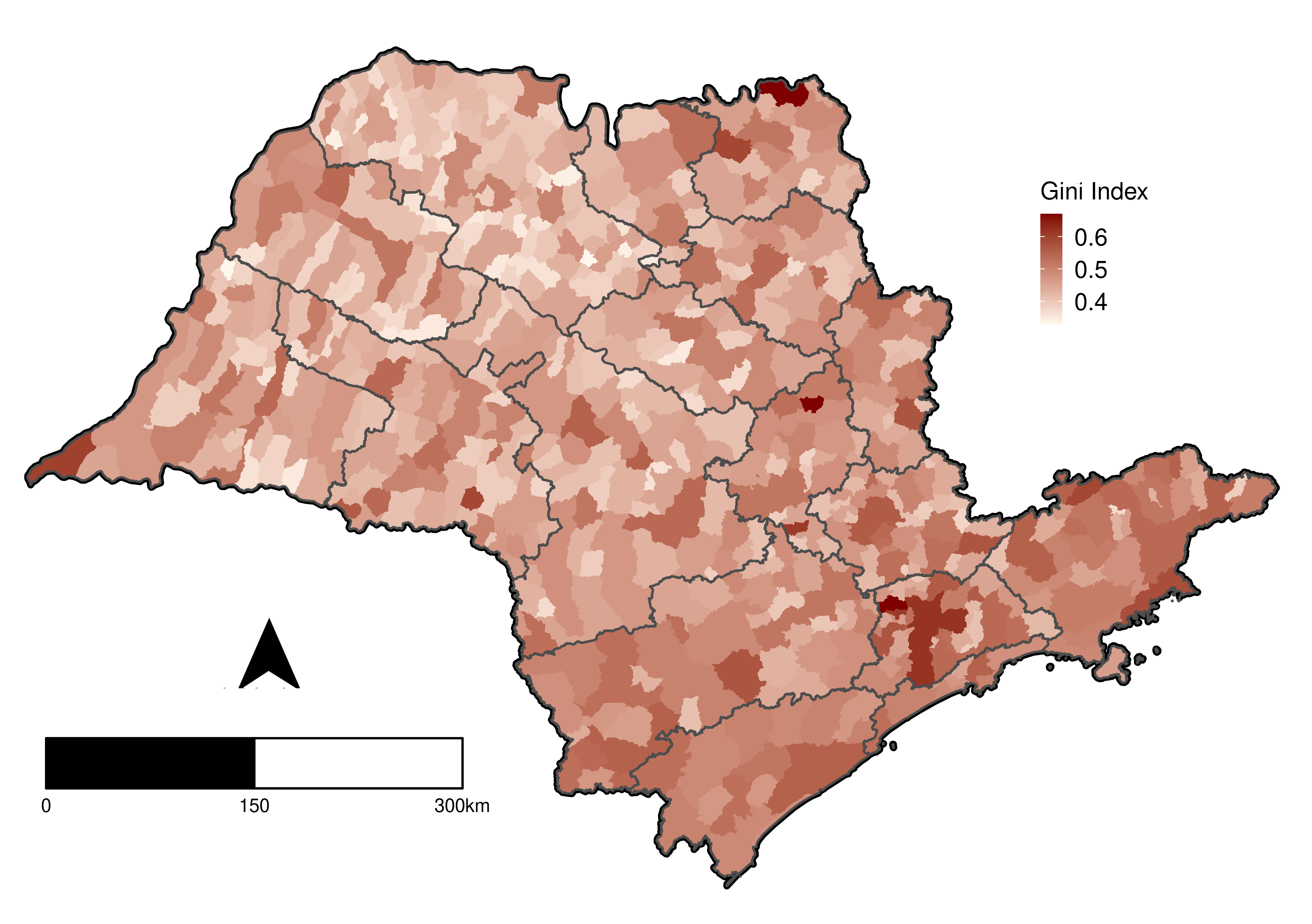

Supplement: S3 Fig — Gini index of the municipalities of the state of Sao Paulo, 2010. Base layer of map: https://www.ibge.gov.br/geociencias/organizacao-do-territorio/malhas-territoriais/15774-malhas.html?=&t=acesso-ao-produto. (PNG) [file pntd.0011435.s008.png]
